# Kinetics of endothelin-1 and effect selective ET_A_ antagonism on ET_B_ activation: a mathematical modeling analysis

**DOI:** 10.3389/fphar.2024.1332388

**Published:** 2024-11-26

**Authors:** K. Melissa Hallow, Peter J. Greasley, Hiddo J. L. Heerspink, Hongtao Yu

**Affiliations:** ^1^ School of Chemical, Materials, and Biomedical Engineering, University of Georgia, Athens, GA, United States; ^2^ Department of Epidemiology and Biostatistics, University of Georgia, Athens, GA, United States; ^3^ Early Clinical Development, Research and Early Development, Cardiovascular, Renal and Metabolism (CVRM), Biopharmaceuticals, R&D, AstraZeneca, Gothenburg, Sweden; ^4^ Department of Clinical Pharmacy and Pharmacology, University of Groningen, Groningen, Netherlands; ^5^ The George Institute for Global Health, Sydney, Australia; ^6^ Clinical Pharmacology and Quantitative Pharmacology, Clinical Pharmacology and Safety Sciences, R&D, AstraZeneca, Gaithersburg, MD, United States

**Keywords:** endothelin, endothelin receptor antagonist, mathematical modeling, kinetics, ET_A_, ET_B_

## Abstract

**Introduction:**

Endothelin-1 (ET-1) regulates renal and vascular function, but the clinical utility of selective ET_A_ receptor antagonists has been limited due to associated fluid retention. The mechanisms underlying fluid retention remain poorly understood but could be a consequence of changes in ET-1 binding to the unantagonized ET_B_ receptor, either through increased ET-1 or non-selective ET_B_.

**Methods:**

A mathematical model of ET-1 kinetics was developed to quantify effects of ET_A_ antagonist exposure and selectivity on concentrations of ET-1 and its complexes with ET_A_ and ET_B_ receptors. The model describes ET-1 production, tissue and plasma distribution, ET_A_ and ET_B_ receptor binding, and receptor-mediated clearance, and was calibrated and validated with human ET-1 infusion studies.

**Results:**

The model confirmed the significant role of ET_B_ in ET-1 clearance. By varying both drug ET_A_ selectivity (K_ib_/K_ia_) and concentration over a wide range, simulations predicted that while selective ET_A_ antagonist (selectivity >1) always decreased [ET1-ET_A_], the change in [ET1-ET_B_] was more complex. It increased up to 45% as drug concentrations approached and exceeded K_ia_, but the increase was diminished as drug concentration increased further and fell below baseline at high concentrations. The drug concentration required to cause a decrease in [ET1-ET_B_] was lower as ET_A_ selectivity decreased.

**Discussion:**

This is the first mechanistic mathematical model of ET-1 kinetics that describes receptor-mediated clearance, and the consequence of ET_B_ blockade on ET-1 concentrations. It provides a useful tool that can coupled with experimental studies to quantitively understand and investigate this complex and dynamic system.

## 1 Introduction

Endothelin-1 (ET-1) is an autocrine/paracrine regulator of renal and vascular function, and antagonism of ET-1 effects has been pursued as a therapeutic target for cardiovascular diseases. ET-1 antagonists have proven beneficial in treating pulmonary arterial hypertension (PAH) (Correale et al., 2018), and been shown to reduce proteinuria and potentially improve outcomes in patients with diabetic kidney disease (DKD) (de Zeeuw et al., 2014; Heerspink et al., 2019). However, their utility in treating cardiovascular diseases has been limited by adverse events related to fluid retention (Packer et al., 2017; Waijer et al., 2021).The mechanisms underlying this effect have proven difficult to fully understand, in part because of the complex physiology of the endothelin system.

ET-1 is produced primarily in the kidney and lungs by conversion of its precursor Big-ET through endothelin converting enzyme (ECE) in endothelial cells. It elicits its physiological effects by binding to two receptors: ET_A_ and ET_B_. It is also cleared by receptor binding, primarily by ET_B_. Binding to ET_A_ mediates vasoconstriction, while ET_B_ is thought to mediate vasodilation and natriuresis. See Davenport et al. (2016) for a thorough review of endothelin physiology.

Endothelin receptor antagonists vary in their selectivity for ET_A_ and ET_B_ receptors. Inhibiting one receptor can cause ET-1 to increase (since clearance is reduced), and thus may increase binding through the other receptor. Because ET_B_ is largely responsible for ET-1 clearance, ET_B_ inhibition in particular may result in a rise in ET-1 binding to ET_A_ (Kelland et al., 2010).

Fluid retention effects of selective ET_A_ antagonists have been proposed to be related to non-selective inhibition of ET_B_ at high doses (Vercauteren et al., 2017; Battistini et al., 2006) or to incompletely understand the pleiotropic effects of ET_A_. A better understanding of ET-1 kinetics and dynamics may aid in the identification of optimal dosing of endothelin antagonists that could provide efficacy while minimizing potential risk of adverse effects.

Understanding the physiological response to endothelin antagonists depends on understanding the degree of inhibition and/or activation of each receptor type. In this study, we developed a mechanistic mathematical model of ET-1 kinetics and blockade by selective or non-selective receptor antagonists. We then utilized this model to quantify the effect of endothelin antagonist selectivity on concentrations of ET-1 to the ET_A_ and ET_B_ receptors in the plasma and tissue compartments. This is a first step in developing a more quantitative understanding of the mechanisms underlying clinically observed responses to endothelin antagonism.

## 2 Methods

### 2.1 Model description

**FIGURE 1:**
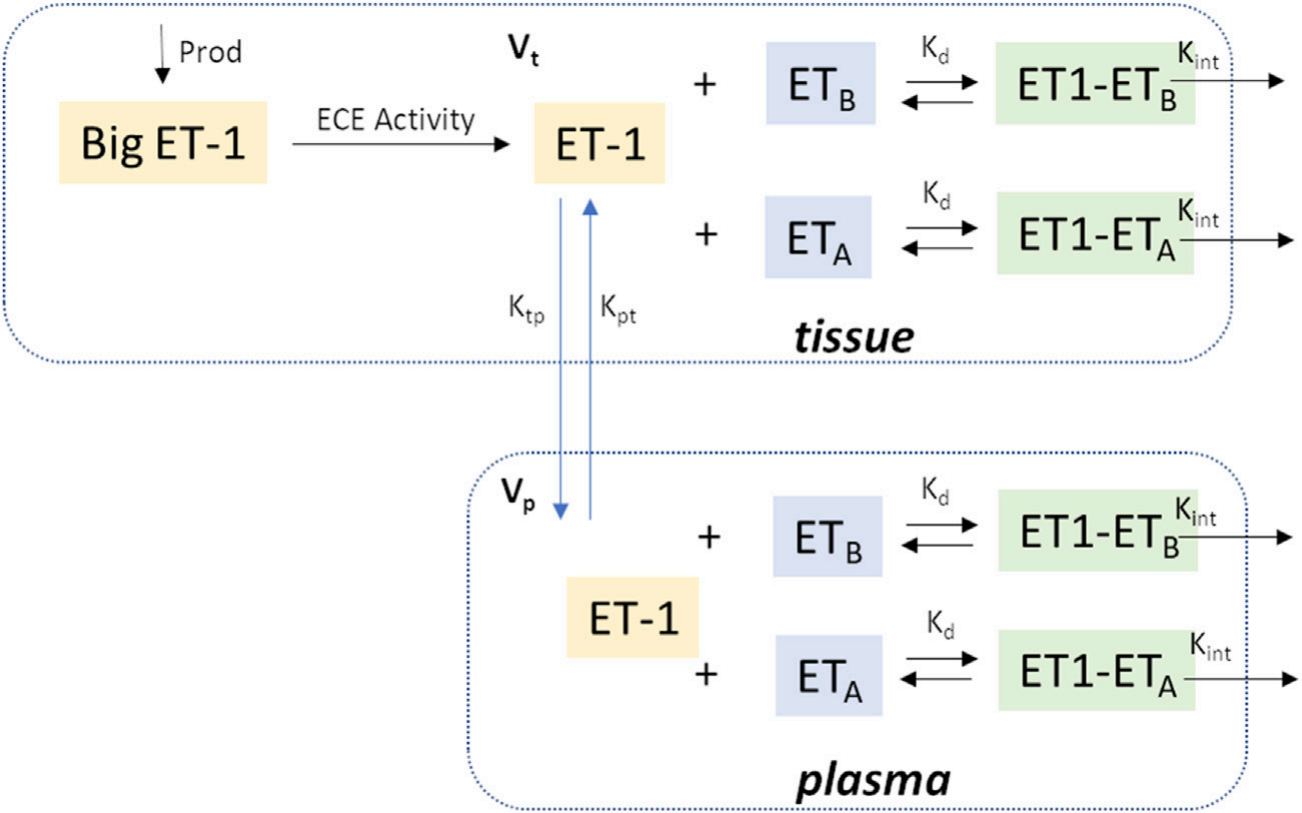
Model Schematic. In brief, Big ET-1 is assumed to be produced at a constant rate; ECE converts Big ET-1 to ET-1 in the tissue compartment; ET-1 is distributed between the tissue and plasma compartments; in each compartment, ET-1 binds to ET_A_ and ET_B_ receptors to form receptor-ligand complexes which are then cleared by internalization. Vp: Central compartment volume; Vt: Tissue compartment volume.

Figure 1 shows a schematic of the ET-1 kinetics model. Big ET-1, the precursor to ET-1, is assumed to be produced endogenously at a constant rate (Prod_BigET_), and is converted to ET-1 through the action of endothelin converting enzyme (ECE).

$$\frac{d\left(\left[BigET\right]\right)}{dt}=Prod_{BigET}-\frac{K_{cat}}{K_{m}}\left[BigET\right]\left[ECE\right]$$ (1)

K_cat_/K_m_ is the catalytic efficiency of ECE (Schweizer et al., 1997).

ET-1 exhibits saturable, high-affinity binding to ET_A_ and ET_B_ receptors, with similar dissociation constant K_d_ for both receptor types (Bacon et al., 1996). ET-1 is cleared by binding to and internalization of these receptors, with most of the clearance occurring through ET_B_. Total ET-1 concentration ([ET1]_tot_) is the sum of concentrations of unbound ET-1 ([ET1]) and ET-1 bound to the ET_A_ and ET_B_ receptors ([ET1-ET_A_] and [ET1-ET_B_],respectively). Because the dissociation constant is similar for both receptors, we lump ET_A_ and ET_B_ receptors together as one receptor concentration [ET1-R] for now. Later, we will revisit this and distinguish between binding to the two receptor types.

$$\left[E{\left.T1\right]}_{tot}=\left[ET1\right]+\left[ET1–{ET}_{A}\right]+\left[ET1{–ET}_{B}\right]=\left[ET1\right]+[ET1–R\right]$$ (2)

Similarly, the total receptor concentration ([R]_tot_) is the sum of free ET_A_ and ET_B_ receptors concentration ([ET_A_] and [ET_B_]), and the ligand-receptor complexes ([ET1-ET_A_] and [ET1-ET_B_]):

$${\left[R\right]}_{tot}=\left[{ET}_{A}\right]+\left[ET{1–ET}_{A}\right]+\left[{ET}_{B}\right]+\left[{ET1–ET}_{B}\right]=\left[R\right]+\left[ET1–R\right]$$ (3)

Receptor binding is assumed to occur several orders of magnitude faster than production, distribution, or internalization, so that equilibrium between binding and dissociation is achieved almost instantaneously, and the ligand, receptor, and ligand-receptor complex are assumed to be in quasi-equilibrium (Mager and Krzyzanski, 2005), so that:

$$K_{d}=\frac{k_{off}}{k_{on}}=\frac{\left[R\right]*\left[ET1\right]}{\left[ET1–R\right]}$$ (4)

Combining Equations 2–4 gives:

$$K_{d}=\frac{\left(R_{tot}-\left({\left[ET1\right]}_{tot}-\left[ET1\right]\right)\right)*\left[ET1\right]}{\left[E{\left.T1\right]}_{tot}-[ET1\right]}$$ (5)

Unbound ET-1 can then be solved from Equation 5 in terms of total ET-1 concentration, total receptor concentrations, and K_d_, as expressed in Equation 6.

$$\left[ET\right]=\left(\frac{1}{2}\right)\left[\left({\left[ET\right]}_{tot}-{\left[R\right]}_{tot}-K_{d}\right)+\sqrt{{\left({\left[ET\right]}_{tot}-{\left[R\right]}_{tot}-K_{d}\right)}^{2}+4K_{d}{\left[ET\right]}_{tot}}\right]$$ (6)

Combining Equations 2, 4 and rearranging, the receptor-ligand complex concentration [ET1-R] is given by:

$$\left[ET1–R\right]=\frac{{\left[R\right]}_{tot}\left[ET1\right]}{K_{d}+\left[ET1\right]}$$ (7)

Most ET-1 production occurs in the lung and kidney, where the highest concentrations of ECE are found (Hunter et al., 2017). Studies of radiolabeled ET-1 have also shown that ET-1 is rapidly cleared from the circulation and taken up in the lungs, kidneys, and liver (Fukuroda et al., 1994; Parker et al., 1999). Thus, ET-1 kinetics are modeled with 2 compartments–a plasma and a tissue compartment. ET-1 production is assumed to be much larger in the tissue than plasma compartment, so that plasma ET-1 production is negligible. For each compartment, the rate of change of total ET-1 is the net sum of ET1 production (tissue compartment only), distribution, and internalization by receptor binding. Total ET-1 in each compartment (*p* denotes plasma and *t* denotes tissue), is given by:

$$\frac{d\left({\left[ET1\right]}_{total,t}\right)}{dt}=Prod_{ET-1}-K_{tp}{\left[ET1\right]}_{t}+K_{pt}{\left[ET1\right]}_{p}-K_{\mathrm{int}}\frac{{\left[R\right]}_{tot,t}{\left[ET1\right]}_{t}}{K_{d}+{\left[ET1\right]}_{t}}$$ (8)

$$\frac{d\left({\left[ET1\right]}_{total,p}\right)}{dt}=K_{tp}{\left[ET1\right]}_{t}-K_{pt}{\left[ET1\right]}_{p}-K_{\mathrm{int}}\frac{{\left[R\right]}_{tot,p}{\left[ET1\right]}_{p}}{K_{d}+{\left[ET1\right]}_{p}}$$ (9)

At steady state, [ET1]_p_ is the normal plasma ET-1 concentration ([ET1]_p0_). There are 7 unknown parameters: the intercompartmental distribution rates K_tp_ and K_pt_, the receptor-ligand internalization rate constant K_int_, the receptor concentrations in each compartment [R]_tot,t_ and [R]_tot,p_, BigET-1 production rate Prod_BigET_, and the concentration of endothelin converting enzyme [ECE].

Endogenous big-ET production is assumed to be constant, and $Prod_{BigET}$ as expressed in Equation 10, can be determined from the steady-state constraint for Equation 1:

$$Prod_{BigET}=\frac{K_{cat}}{K_{m}}{\left[BigET\right]}_{0}\;*\left[ECE\right]$$ (10)

The steady-state tissue concentration of ET-1 can be determined from Equation 9 at steady-state:

$${\left[ET1\right]}_{t0}=\frac{K_{\text{pt}}{\left[ET1\right]}_{p0}+K_{\mathrm{int}}{\left[R\right]}_{tot,p}\frac{{\left[ET1\right]}_{p0}}{K_{d}+{\left[ET1\right]}_{p0}}}{K_{tp}}$$ (11)

Then, the total tissue receptor concentration (Equation 12), which is assumed constant, can be determined from Equation 8 at steady-state and Equation 11.

$${\left[R\right]}_{tot,t}=\frac{Prod_{ET-1}-K_{tp}{\left[ET1\right]}_{t0}+K_{pt}{\left[ET1\right]}_{p0}}{K_{\mathrm{int}}\left(\frac{{\left[ET1\right]}_{p0}}{K_{d}+{\left[ET1\right]}_{p0}}\right)}$$ (12)

This leaves 5 parameters to be estimated by fitting experimental data.

### 2.2 Parameter estimation

Unknown model parameters were estimated by simultaneously fitting three different experimental studies. Each study provided important pieces of information for parameter estimation.

*Radiolabeled ET-1 clearance study:* In Parker et al. (1999), 5 healthy human participants were administered a bolus venous infusion of radiolabeled ET-1 over 5 minutes, and radiolabeled plasma ET-1 was measured at 0, 1, 2, 3, 4, 5, 6, 8, 10, 12, 14, 16, 18, 20, 25, 30, 35, 40, 45, 50, 60, 70, 80, 100, 120, 150, 180, 210 and 240 min after the start of the infusion. This study provided information for constraining intercompartmental distribution and receptor internalization rates. However, the ET-1 dose was unknown and assumed tiny relative to plasma ET-1, so only relative concentrations could be fit.

*Infusion of increasing doses of ET-1:* In Kaasjager et al. (1997), 6 healthy participants were administered an infusion of ET-1 at increasing infusion rates. Participants received 0.5 ng/kg/min ET-1 for 60 min, followed by 1 ng/kg/min for 60 min, followed by a final 2.0 ng/kg/min for 60 min. Plasma ET-1 was measured before infusion and at 75, 125, and 225 min after the start of the infusion. This study provided further information for constraining intercompartmental distribution and receptor internalization rates, and also provided information for constraining receptor concentration and compartment volumes.

*Infusion of Big ET-1:* In Hunter et al. (2017), 10 healthy human participants were administered an infusion of Big-ET at increasing infusion rates. Participants received 0.75 pmol/min for 30 min, followed by 15 pmol/min for 30 min, followed by 300 pmol/min for another 30 min. Plasma ET-1 was measured at baseline and at 30-min intervals through 150 min. This study provided information for quantifying ECE concentration, and further information for constraining intercompartmental distribution rates, volumes, and receptor concentration.

Fitting these three studies simultaneously provided sufficient information to estimate all model parameters. The study protocol for each study was simulated. Parameters were estimated by minimizing the least square error between the observed and model-predicted plasma ET-1 concentrations.

### 2.3 Distinguishing ET_A_ and ET_B_ binding and internalization

After estimating model parameters with lumped ET_A_ and ET_B_, we then separated out the contributions of ET1_A_ and ET1_B_.

Let f_B_ be the fraction of total receptors that are ET_B_ receptors. Then the fraction of total receptors that are ET_A_ receptors, f_A_, is 1 – f_B_.

Then, the concentration of each receptor (in the absence of an inhibitor) can be determined, as given in Equations 13, 14:

$${{\left[R_{B}\right]}_{tot}=f}_{B}{\left[R\right]}_{tot}$$ (13)

$${\left[R_{A}\right]}_{tot}=\left({1-f}_{B}\right){\left[R\right]}_{tot}$$ (14)

And concentration of the bound complex can then be expressed as Equations 15, 16:

$$\left[ET1–R_{A}\right]=\frac{{\left[R_{A}\right]}_{tot}\left[ET1\right]}{K_{d}+\left[ET1\right]}$$ (15)

$$\left[E{T1–R}_{B}\right]=\frac{{\left[R_{B}\right]}_{tot}\left[ET1\right]}{K_{d}+\left[ET1\right]}$$ (16)

The relative expression of ET_A_ and ET_B_ receptors differ across tissues. The density of ET_A_ is much higher than ET_B_ in resistance vessels. In the lung, which is the tissue with the highest overall receptor concentration, the fraction of ET_B_ is around 40%, while in the kidney it is around 70%–80% (Davenport et al., 2016; Kuc et al., 1995). Thus, we allow f_B_ to be estimated separately for tissue and plasma compartments.

Equations 8, 9 can be rewritten to Equations 17, 18 as:

$$V_{t}\frac{d\left({\left[ET1\right]}_{tot,t}\right)}{dt}=Prod_{ET-1}-K_{tp}V_{t}{\left[ET1\right]}_{t}+K_{pt}V_{p}{\left[ET1\right]}_{p}-K_{\mathrm{int}}V_{t}\left({\left[R_{A}\right]}_{tot,t}+{\left[R_{B}\right]}_{tot,t}\right)\;\frac{{\left[ET1\right]}_{t}}{K_{d}+{\left[ET1\right]}_{t}}$$ (17)

$$V_{p}\frac{d\left({\left[ET1\right]}_{tot,p}\right)}{dt}=K_{tp}V_{t}{\left[ET1\right]}_{t}-K_{pt}V_{p}{\left[ET1\right]}_{p}-K_{\mathrm{int}}V_{p}\left({\left[R_{A}\right]}_{tot,p}+{\left[R_{B}\right]}_{tot,p}\right)\frac{{\left[ET1\right]}_{p}}{K_{d}+{\left[ET1\right]}_{p}}$$ (18)

### 2.4 Modeling competitive ET_A_ and ET_B_ inhibition

Endothelin antagonists are competitive inhibitors with varying degrees of selectivity for ET_A_ or ET_B_ receptors. Let [I] be the concentration of a competitive endothelin antagonist, with an affinity K_ia_ for ET_A_ receptors and K_ib_ for ET_B_ receptors. The concentration of the ligand-receptor complex in the presence of an antagonist can be expressed as Equations 19, 20 (see Supplementary Material for derivation):

$$\left[ET1–R_{A}\right]=\frac{{\left[R_{A}\right]}_{tot}\left[ET1\right]}{K_{d}\left(1+\frac{\left[I\right]}{K_{ia}}\right)+\left[ET1\right]}$$ (19)

$$\left[ET1–R_{B}\right]=\frac{{\left[R_{B}\right]}_{tot}\left[ET1\right]}{K_{d}\left(1+\frac{\left[I\right]}{K_{ib}}\right)+\left[ET1\right]}$$ (20)

It can further be shown that the concentrations of free ET_A_ and ET_B_ receptors are:

$$\left[R_{A}\right]=\frac{{\left[R_{A}\right]}_{tot}}{1+\frac{\left[ET1\right]}{K_{d}}+\frac{\left[I\right]}{K_{ia}}}$$ (21)

$$\left[R_{B}\right]=\frac{{\left[R_{B}\right]}_{tot}}{1+\frac{\left[ET1\right]}{K_{d}}+\frac{\left[I\right]}{K_{ib}}}$$ (22)

Substituting Equations 21, 22 into Equation 2 gives ET1_tot_, as expressed in Equation 23.

$$E{T1}_{tot}=\left[ET1\right]+\frac{\left[ET1\right]}{K_{d}}\left(\;\frac{{\left[R_{A}\right]}_{tot}}{1+\frac{\left[ET1\right]}{K_{d}}+\frac{\left[I\right]}{K_{ia}}}+\frac{{\left[R_{B}\right]}_{tot}}{1+\frac{\left[ET1\right]}{K_{d}}+\frac{\left[I\right]}{K_{ib}}}\right)$$ (23)

With some additional algebra, the concentration of free [ET1] can be obtained by solving the resulting third order polynomial for [ET1] (see Supplementary Material for full derivation).

### 2.5 Validation

To validate the model, a separate experimental study, not used in model calibration, was simulated and compared with study results.

Validation Dataset: ET_A_ or ET_B_ inhibition followed by ET-1 infusion: In Bohm et al. (2003), 6 healthy, male participants were studied on 3 different days separated by at least 1 week. Participants were infused with either 0.9% saline (for 15 min), the ET_A_ inhibitor BQ123 (2.5–5 nmol/kg/min for 50 min), or the ET_B_ inhibitor BQ788 (4 nmol/kg/min for 15 min). After 30 min, participants were also infused with ET-1 (4 pmol/kg/min) for 20 min. Plasma ET-1 was measured at 0, 15, 30, 40, and 50 min.

To model this study, binding affinities and selectivity of the selective ET_A_ antagonist BQ123 and selective ET_B_ antagonist BQ788 were set to previously reported values in human tissue (BQ123: K_ia_ = 0.78 nM, K_ib_ = 24.3 μM (Peter and Davenport, 1996); BQ788: K_ia_ = 1 μM, K_ib_ = 9.8 nM (Russell and Davenport, 1996)).

### 2.6 Sensitivity analysis

To evaluate which parameters contribute most to the uncertainty in the model output, we computed the Sobol indices using the sensobol package in R (Puy et al., 2022), a form of global sensitivity analysis (IM SJMMCE, 1993). Assuming mutual independence among the input parameters, the variance of the output is decomposed into fractions which can be attributed either to a single input parameter (first order Sobol indices) or to a set of parameters (higher order Sobol indices). The total-order index T_i_ measures the first-order effect of a parameter jointly with its interactions with all the parameters (Homma and Saltelli, 1996).

### 2.7 Model implementation

The model was implemented in R v4.1.2 using the RxODE package (Wang et al., 2016). Optimization was performed using the L-BFGS-B method in the optim package. Model code is available at https://bitbucket.org/cardiorenalmodel/endothelin-kinetics.

## 3 Results and discussion

### 3.1 Model calibration

**FIGURE 2:**
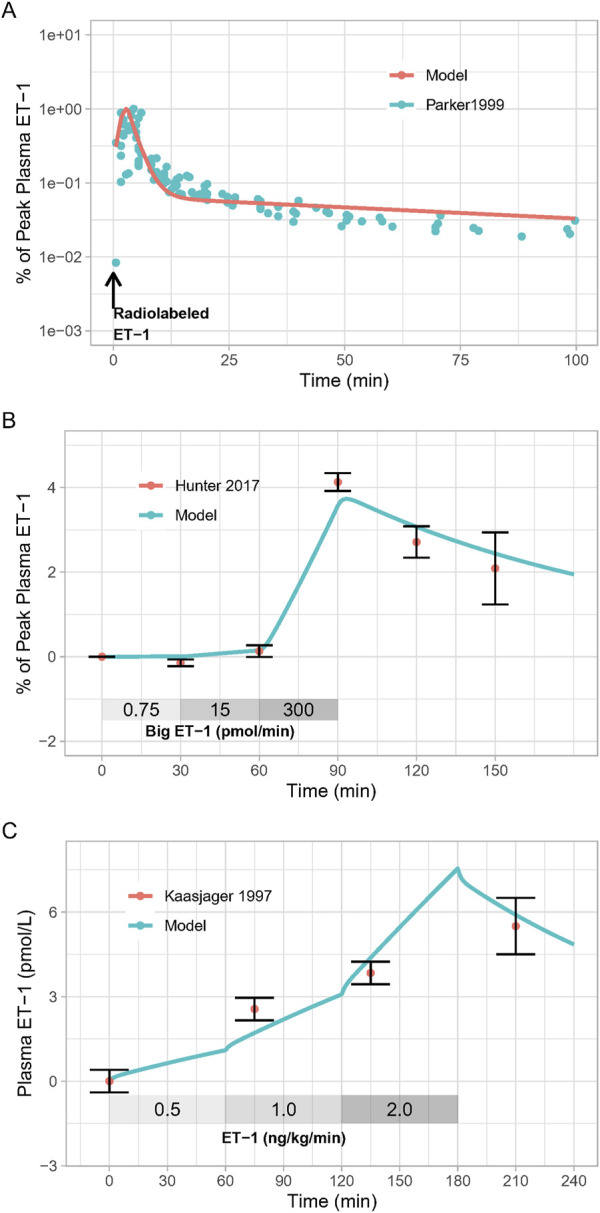
Model Calibration: Model parameters were estimated by fitting: **(A)**, the response to radiolabeled ET-1 bolus (Parker et al., 1999); **(B)**, increasing doses of Big ET-1 infusion (Hunter et al., 2017); **(C)**, increasing doses of ET-1 infusion (Kaasjager et al., 1997).

As shown in Figure 2, the calibrated model reasonably reproduced the observed magnitude and time-course of changes in ET-1 following an ET-1 bolus (Figure 2A), increasing rates of Big ET-1 infusion (Figure 2B), or increasing rates of ET-1 infusion (Figure 2C). Estimated parameter values are given in Table 1. In order to simultaneously fit all three studies, it was necessary to allow [ECE] to vary for each study. For all other estimated parameters, the same estimated values allowed the model to reasonably fit all studies simultaneously. Simultaneously fitting all studies did require some trade-off in fit: each study could be fit more precisely if parameters were estimated separately for each study. However, the simultaneously fit parameters are more useful than study-specific parameters in providing a general model of ET-1 kinetics, and thus these parameters were used for the rest of this analysis.

**TABLE 1:** Model parameters.

Parameter	Definition	Value	Units	Source
BigET(0)	Normal plasma Big ET-1 concentration, initial condition	0.93	pmol/L	Miyauchi et al. (2012)
[ET1]_p_(0)	Normal plasma ET-1 concentration, initial condition	3.2	pmol/L	Kaasjager et al. (1997)
K_cat_/K_m_	ECE catalytic efficiency	2.64e-4	L/min/pmol	Schweizer et al. (1997)
K_d_	ET-1 dissociation constant for ET_A_ and ET_B_	400	pmol/L	Bacon et al. (1996)
V_p_	Central compartment volume	81.6 (1.1%)	L	estimated
V_t_	Tissue compartment volume	2.64 (7%)	L	estimated
[ECE]	Endothelin converting enzyme concentration	Parker: 162.6 (2.5%) Hunter: 98 (4.6%) Kaasjager: 27 (10.7%)	nmol/L	estimated
K_pt_	ET-1 distribution rate from plasma to tissue	0.87 (18.5%)	/min	estimated
K_tp_	ET-1 distribution rate from tissue to plasma	0.98 (2.3%)	/min	estimated
K_int_	Receptor-ligand internalization rate	0.0095 (0.4%)	pmol/min	estimated
R_tot,p_	Total receptor concentration in plasma compartment	460 (1.2%)	pmol/L	estimated
R_tot,t_	Total receptor concentration in tissue compartment	7,738	pmol/L	Calculated from steady-state constraints
[ET1]_t_(0)	Total (bound and unbound) concentration of ET-1, initial condition	88.3	pmol/L	Calculated from steady-state constraints
f_B,t_	Fraction of total receptors that are ET_B_ receptors in tissue compartment	0.65 (11%)	—	estimated
f_B,c_	Fraction of total receptors that are ET_B_ receptors in plasma compartment	0.8 (15%)	—	estimated

### 3.2 Model Validation

**FIGURE 3:**
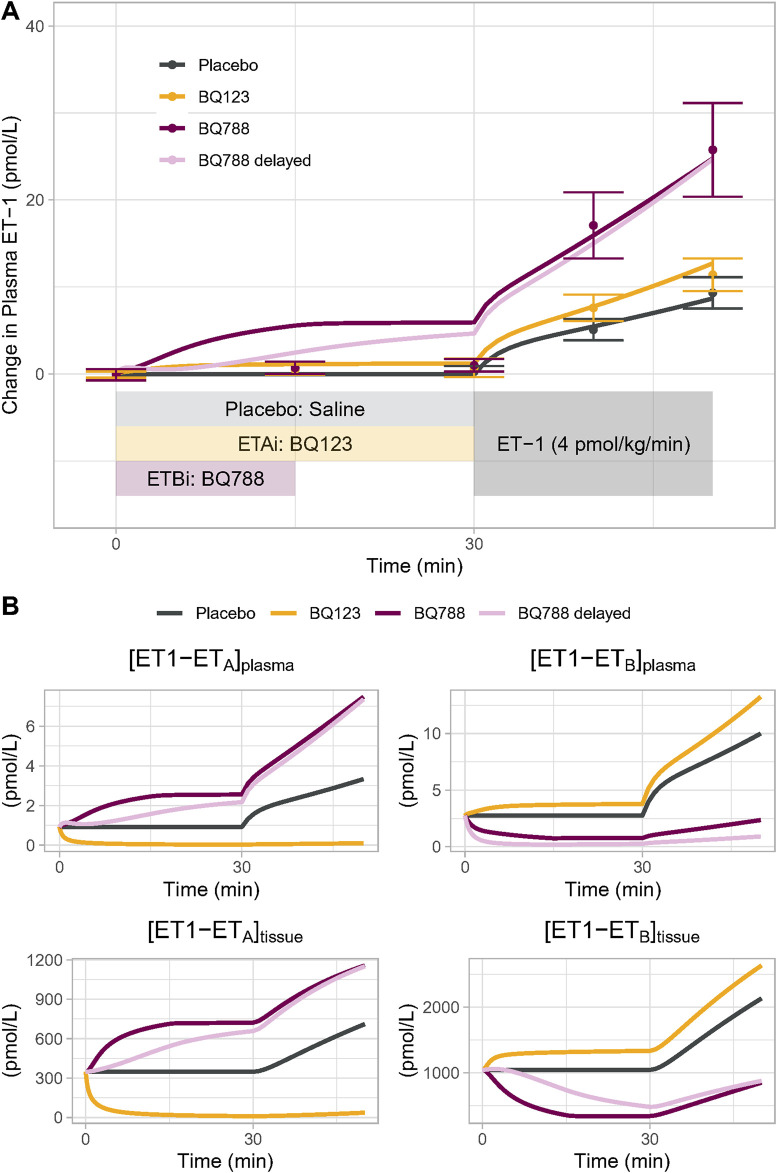
**(A)** Model Validation: The calibrated model reproduced experimentally observed changes in plasma ET-1 observed by Bohm et al. (2003) in response to placebo, BQ123 (ET_A_ antagonist 4 nmol/kg/min for 50 min), or BQ788 (ET_B_ antagonist 4 nmol/kg/min for 15 min) followed by ET-1 infusion. Speed of rise in plasma ET-1 with BQ788 is overpredicted; assuming a delay between plasma drug concentration and tissue inhibitory effect on ET_B_ (light purple) more closely reproduces the data **(B)** Model-predicted changes in the physiologically active bound complexes of ET1 to ET_A_ or ET_B_ in the plasma and tissue compartments.

The calibrated model was able to reproduce the changes in plasma ET-1 observed by Bohm et al. (2003) (Figure 3A). First, the model reproduced the change in plasma ET-1 during ET-1 infusion in the placebo arm, demonstrating that the ET-1 model can predict ET-1 kinetics in a new experiment. Secondly, the model reproduced the augmented rises in ET-1 with selective ET_A_ or ET_B_ antagonist, resulting from reduced clearance when the receptors are inhibited. Consistent with the experimental data, the rise in ET-1 with ET_B_ antagonism was much greater than with ET_A_ antagonism, indicating that the model recapitulates the dominant role of ET_B_ in ET-1 clearance.

For ET_B_ antagonism, the model did overpredict the increase in ET-1 during the period of ET_B_ antagonism alone, prior to the start of ET-1 infusion. While Bohm et al. reported no change in ET-1 during this period, other studies have found that ET-1 does increase with similar doses of BQ788 (Okada and Nishikibe, 2002; Strachan et al., 1999), but this increase is delayed. This could be due to a delay in BQ788 reaching ET_B_ in peripheral tissues. When a pharmacodynamic delay was introduced, the model came closer to reproducing the observed ET-1 changes. Because other studies have noted a rise in ET-1 with BQ788, we did not want to overfit the model to this single datapoint in this single study, and thus no further changes were made to force fit this point.

### 3.3 Simulations

#### 3.3.1 Effect of selective ET receptor antagonism on non-antagonized receptor complex

Changes in ET_B_ activation with selective ET_A_ antagonists have been proposed as a mechanism for fluid retention with ET_A_ receptor antagonists. On one hand, inhibition of ET_B_ at high doses of selective ET_A_ receptor antagonists has been proposed to cause fluid retention by blocking natriuretic/diuretic effects of ET_B_ (Battistini et al., 2006; Baltatu et al., 2012). On the other hand, activation of ET_B_ receptors as a consequence of elevated ET-1 with ET_A_ antagonism has been proposed to increase vascular permeability and redistribute plasma volume, resulting in edema (Vercauteren et al., 2017). A first step in understanding these possible mechanisms is to quantify how the concentration of a selective antagonist affects plasma ET-1 and the formation of bound complex with the non-antagonized receptor.

We first simulated a perfectly selective ET_A_ antagonist by setting K_ia_ to 1 and K_ib_ to 10^20^ (to approximate zero ET_B_ antagonism). The drug concentration was then varied from 0.001 to 1,000X K_ia_, and steady-state changes in the bound complexes [ET1-ET_A_] and [ET1-ET_B_] were determined in the plasma and tissue compartments. This was repeated for a perfectly selective ET_B_ antagonist, with K_ia_ set to 10^20^ (to approximate zero ET_A_ antagonism)and K_ib_ set to 1, and drug concentration varied from 0.001 to 100,000X K_ib_.

**FIGURE 4:**
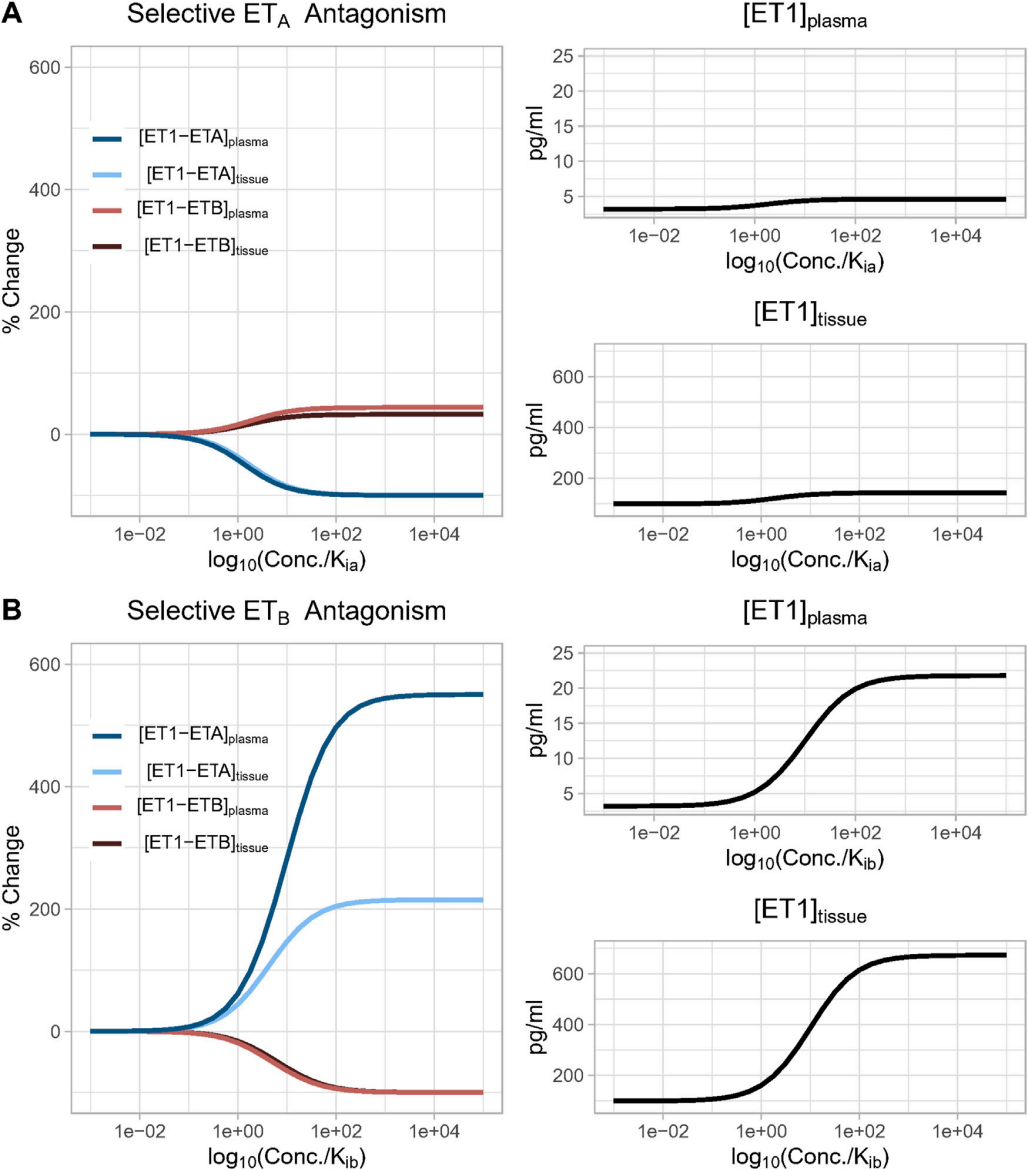
**(A)** Effect of increasing concentration of a perfectly selective ET_A_ antagonist. Simulation predicts that as the concentration of a selective ET_A_ antagonist increases, the formation of bound complex [ET1-ET_B_] increases up to 33% and 45% in the tissue and plasma compartments, respectively, as bound complex [ET1-ET_A_] suppression approaches 100%; **(B)** Effect of increasing concentration of a perfectly selective ET_B_ antagonist. Simulation predicts that as the concentration of a selective ET_B_ antagonist increases, the formation of bound complex [ET1-ET_A_] increases more than 200% and 500% in the tissue and plasma compartments, respectively, as bound complex [ET1-ET_B_] suppression approaches 100%.

As shown in Figure 4A, as the concentration of a selective ET_A_ antagonist was increased relative to K_ia_, the formation of bound complex [ET1-ET_B_] increased up to 33% and 45% in the tissue and plasma compartments, respectively, as bound complex [ET1-ET_A_] suppression approached 100%. For selective ET_B_ antagonism (Figure 4B), as the concentration was increased relative to K_ib_, the rise in ET1-ET_A_ complex was quite large, increasing to more than 200% and 500% in the tissue and plasma compartments, respectively, as bound complex [ET1-ET_B_] suppression approached 100%.

In both cases, the rise in the complex of ET-1 with the non-inhibited receptor occurred due to a compensatory rise in ET-1 concentration, since inhibiting either receptor reduced ET-1 clearance. Since ET_B_ is responsible for a larger portion of ET-1 clearance than ET_A_, the rise in ET-1 with ET_B_ antagonism was much larger than with ET_A_ antagonism. Consequently, the rise in [ET1-ET_A_] with ET_B_ antagonism was also much larger than the rise in ET1-ET_B_ with ET_A_ antagonism.

If there were no change in ET-1 concentration, it would be expected that when the drug concentration equals K_i_ (when log10(conc/K_i_) = 1), the complex of ET-1 with the antagonized receptor would be reduced 50%. However, in both cases, the concentration required to produce a 50% reduction was shifted higher as a result of the rise in ET-1 concentration (See Equations 19, 20). This shift was much larger with ET_B_ antagonism, due to the larger rise in ET-1.

Sobol sensitivity analysis indicated that the uncertainty in predicted changes in ET1-ET_A_ or ET1-ET_B_ was nearly completely due to the choice of f_B_–fraction of total receptors that are ET_B_ receptors. To explore the effect of f_B_ on the model predictions, we repeated the simulations above when f_B_ is set to 0.5 (a scenario of equal concentrations of ET_A_ and ET_B_ receptors, and thus equal clearance through each receptor–inconsistent with (Bohm et al., 2003) and other studies (Fukuroda et al., 1994; Dupuis et al., 1996)), or to 0.999 (a scenario in which ET receptors are 99.9% ET_B_ and 0.1% ET_A_). In the first case, the rise in the non-antagonized receptor complex was equal for selective ET_A_ and ET_B_ antagonists (i.e., ET1-ET_B_ rise with ET_A_ antagonism was the same as ET1-ET_A_ rise with ET_B_ antagonism). The ET-1 concentration also rose equally. At the other extreme, when f_B_ is set to 0.999, there was no change in ET1-ET_B_ with ET_A_ antagonism, but ET1-ET_A_ increased more than 2000-fold with ET_B_ antagonism. However, in all cases, the shape of the curves, and thus the dependency on K_i_ and concentration, remained the same. Only the magnitudes changed (Supplementary Figures S1, S2).

#### 3.3.2 Effect of antagonist selectivity on non-antagonized receptor complex

**FIGURE 5:**
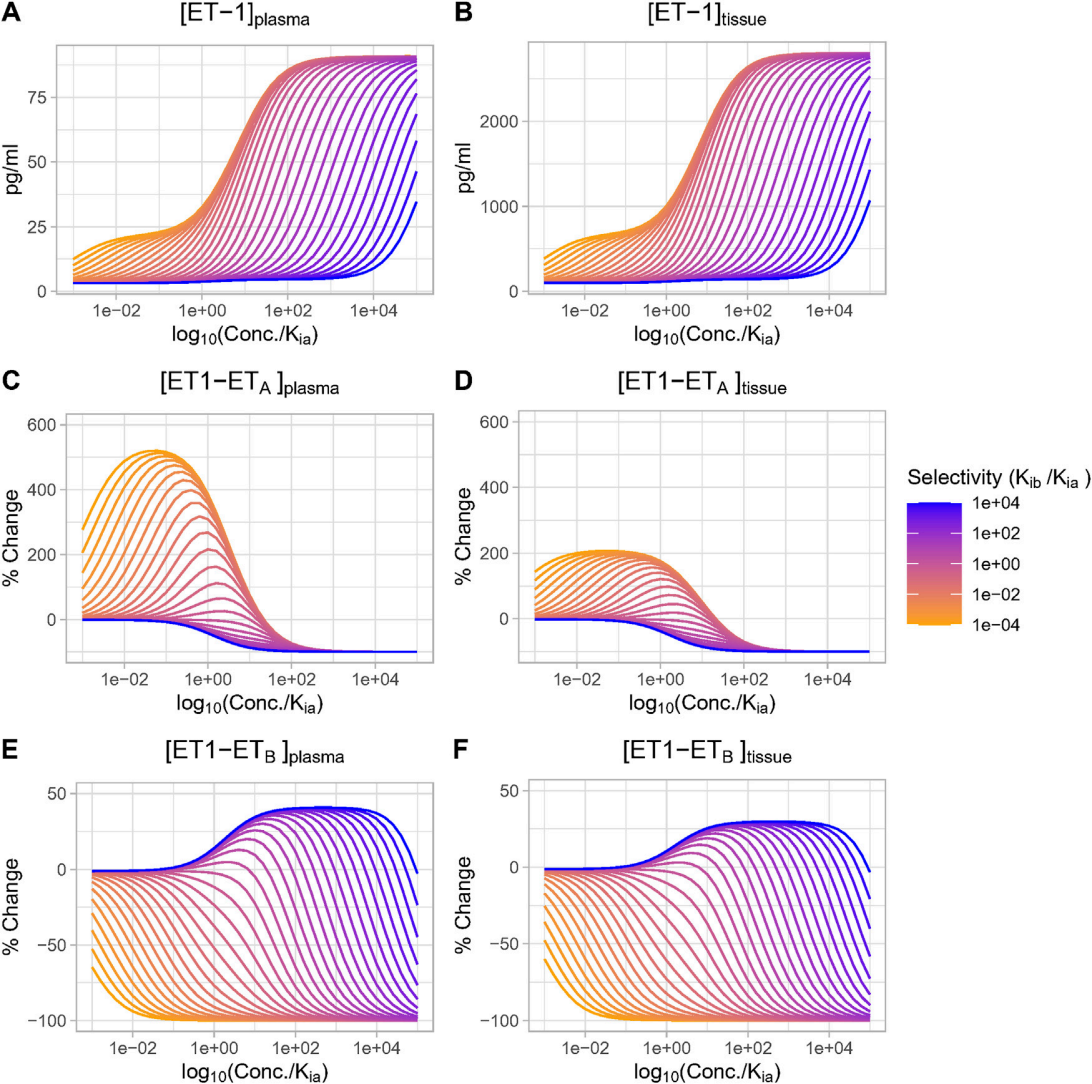
Effect of antagonist selectivity on plasma and tissue changes in ET-1 **(A, B)**, ET_A_ activation by ET-1 **(C, D)**, ET_B_ activation by ET-1 **(E, F)**. ET_A_ antagonism: selectivity >1, ET_B_ antagonism: selectivity <1.

We then investigated the effect of antagonist receptor selectivity by varying both drug ET_A_ selectivity (K_ib_/K_ia_) and drug concentration over a wide range. In Figure 5, all concentrations are plotted relative to K_ia_ for consistency. [ET-1] increased with increasing concentrations for all selectivity values, but the higher the selectivity for ET_A_, the higher the drug concentration (relative to K_ia_) required to increase ET-1 (Figures 5A, B). Trends were the same but concentrations were much higher in the tissue compared to plasma.

The complex [ET1-ET_A_] always decreased with increasing concentration of selective ET_A_ antagonist (selectivity >1). For ET_B_ selective antagonism (selectivity <1), [ET1-ET_A_] was non-monotonic–for concentrations well below Kia, it increased, and increased faster with increasing. However, as concentrations approached and exceeded K_ia_ (and thus also far exceeded K_ib_), the rise in [ET1-ET_A_] began to become smaller, and [ET1-ET_A_] eventually began to decrease at concentrations well above K_ia_ (Figures 5C, D).

The complex [ET1-ET_B_] always decreased with increasing concentrations of ET_B_-selective antagonists (selectivity <1). Interestingly, though, for ET_A_-selective antagonists, the rise in [ET1-ET_B_] was minimal at concentrations less than 0.1X K_ia_, then became larger as concentrations approached and exceeded K_ia_. After reaching a maximum increase of around 45% (plasma) or 33% (tissue), further increases in concentration did not further increase [ET1-ET_B_]. Instead, as concentrations rose further, [ET1-ET_B_] began to fall and quickly became negative. The concentration required to cause a decrease in [ET1-ET_B_] was higher as selectivity increased (Figures 5E, F).

Thus, depending on the concentration, ET_A_ antagonists can increase (at low concentrations) or decrease (at high concentrations) the activation of ET_B_. The higher the selectivity for ET_A_, the higher the concentration required to cause ET_B_ to decrease.

**FIGURE 6:**
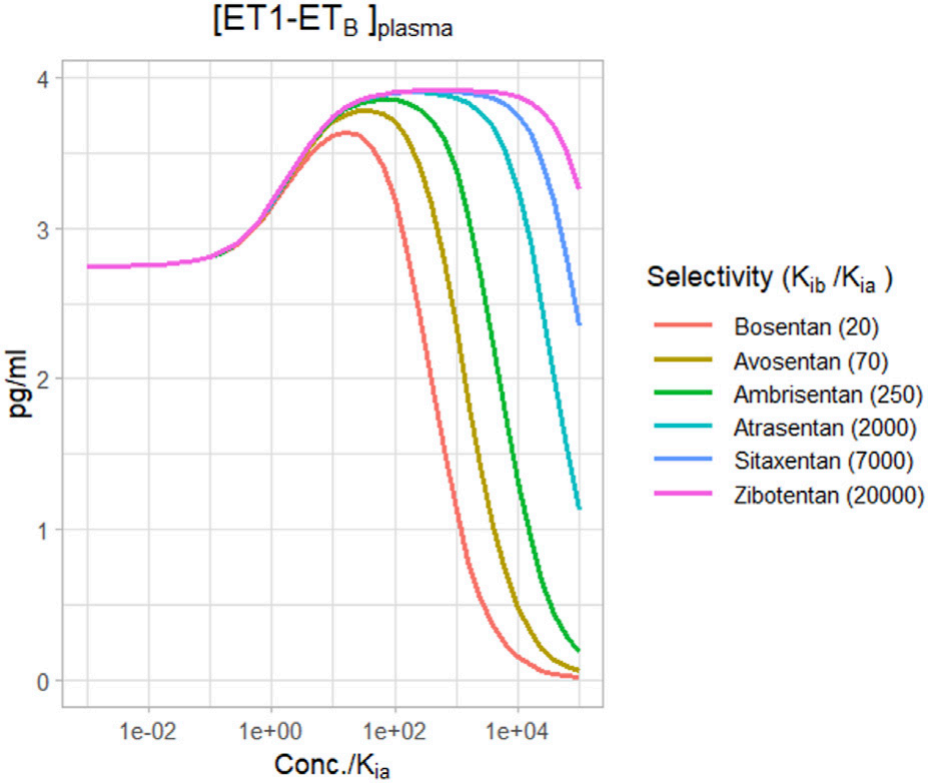
Effect of ET_A_ antagonists with varying degrees of selectivity on ET_B_ activation by ET1.

Figure 6 shows the change in plasma [ET1-ET_B_] for different selective ET_A_ antagonists, based on their reported selectivities (Davenport et al., 2016). For a relatively non-selective antagonist like bosentan, [ET1-ET_B_] rise did not quite reach the maximum before falling, and became negative at concentrations around 100X K_ia_. However, for more selective ET_A_ antagonists, the rise in [ET1-ET_B_] tended to max out as concentrations rose. There was no difference in the maximum rise between ambrisentan, atrasentan, sitaxentan, and zibotentan. However, while ambrisentan causes [ET1-ET_B_] to become negative at concentrations around 1,000x K_ia_, [ET1-ET_B_] remained positive with zibotentan for concentrations up to 100,000xK_ia_.

Several limitations should be noted. Receptor concentrations of ET_A_ and ET_B_ vary across tissues and across species. This analysis assumed a constant relative concentration of receptors, but this could vary by tissue. Receptor concentration may also change due to compensatory upregulation or downregulation due to antagonism, and this was not considered. Nearly all of the experimental data used to develop the model was collected in males, and there are likely sex differences that could impact the model’s predictiveness in females. Endogenous ET-1 production was assumed constant, but in reality its secretion changes in response to physiological signals.

## 4 Conclusion

This is the first mechanistic mathematical model of ET-1 kinetics that describes receptor-mediated clearance, and the consequence of ET_B_ blockade on ET-1 concentrations. It provides a useful tool that can coupled with experimental studies to quantitively understand and investigate this complex and dynamic system. This analysis quantifies effect of ET_A_ antagonists on ET_B_ activation, but does not describe the physiological consequences of changes in ET_A_ and ET_B_ binding. This is addressed in our sister paper.

## Data Availability

Publicly available datasets were analyzed in this study. This data can be found here: https://bitbucket.org/cardiorenalmodel/endothelin-kinetics.

## References

[B1] Bacon C. R. Cary N. R. B. Davenport A. P. (1996). Endothelin peptide and receptors in human atherosclerotic coronary artery and aorta. Circulation Res. 79 (4), 794–801. 10.1161/01.res.79.4.794 8831503

[B2] Baltatu O. C. Iliescu R. Zaugg C. E. Reckelhoff J. F. Louie P. Schumacher C. (2012). Antidiuretic effects of the endothelin receptor antagonist avosentan. Front. physiology 3, 103. 10.3389/fphys.2012.00103 PMC3328756 22529820

[B3] Battistini B. Berthiaume N. Kelland N. F. Webb D. J. Kohan D. E. (2006). Profile of past and current clinical trials involving endothelin receptor antagonists: the novel “-sentan” class of drug. Exp. Biol. Med. (Maywood, NJ) 231 (6), 653–695. 10.3181/00379727-231-2310653 16740981

[B4] Bohm F. Pernow J. Lindstrom J. Ahlborg G. (2003). ETA receptors mediate vasoconstriction, whereas ETB receptors clear endothelin-1 in the splanchnic and renal circulation of healthy men. Clin. Sci. (Lond). 104 (2), 143–151. 10.1042/CS20020192 12546636

[B5] Correale M. Ferraretti A. Monaco I. Grazioli D. Di Biase M. Brunetti N. D. (2018). Endothelin-receptor antagonists in the management of pulmonary arterial hypertension: where do we stand? Vasc. Health Risk Manag. 14, 253–264. 10.2147/VHRM.S133921 30323613 PMC6174907

[B6] Davenport A. P. Hyndman K. A. Dhaun N. Southan C. Kohan D. E. Pollock J. S. (2016). Endothelin. Pharmacol. Rev. 68 (2), 357–418. 10.1124/pr.115.011833 26956245 PMC4815360

[B7] de Zeeuw D. Coll B. Andress D. Brennan J. J. Tang H. Houser M. (2014). The endothelin antagonist atrasentan lowers residual albuminuria in patients with type 2 diabetic nephropathy. J. Am. Soc. Nephrol. 25 (5), 1083–1093. 10.1681/ASN.2013080830 24722445 PMC4005314

[B8] Dupuis J. Goresky C. A. Fournier A. (1996). Pulmonary clearance of circulating endothelin-1 in dogs *in vivo*: exclusive role of ETB receptors. J. Appl. physiology (Bethesda, Md 1985) 81 (4), 1510–1515. 10.1152/jappl.1996.81.4.1510 8904561

[B9] Fukuroda T. Fujikawa T. Ozaki S. Ishikawa K. Yano M. Nishikibe M. (1994). Clearance of circulating endothelin-1 by ETB receptors in rats. Biochem. Biophys. Res. Commun. 199 (3), 1461–1465. 10.1006/bbrc.1994.1395 8147891

[B10] Heerspink H. J. L. Parving H. H. Andress D. L. Bakris G. Correa-Rotter R. Hou F. F. (2019). Atrasentan and renal events in patients with type 2 diabetes and chronic kidney disease (SONAR): a double-blind, randomised, placebo-controlled trial. Lancet London, Engl. 393 (10184), 1937–1947. 10.1016/S0140-6736(19)30772-X 30995972

[B11] Homma T. Saltelli A. (1996). Importance measures in global sensitivity analysis of nonlinear models. Reliab. Eng. and Syst. Saf. 52 (1), 1–17. 10.1016/0951-8320(96)00002-6

[B12] Hunter R. W. Moorhouse R. Farrah T. E. MacIntyre I. M. Asai T. Gallacher P. J. (2017). First-in-Man demonstration of direct endothelin-mediated natriuresis and diuresis. Hypertens. (Dallas, Tex 1979) 70 (1), 192–200. 10.1161/HYPERTENSIONAHA.116.08832 PMC5739104 28507171

[B13] IM SJMMCE. (1993). Sensitivity estimates for nonlinear mathematical models. 1(4):407–414.

[B14] Kaasjager K. A. Shaw S. Koomans H. A. Rabelink T. J. (1997). Role of endothelin receptor subtypes in the systemic and renal responses to endothelin-1 in humans. J. Am. Soc. Nephrol. 8 (1), 32–39. 10.1681/ASN.V8132 9013446

[B15] Kelland N. F. Kuc R. E. McLean D. L. Azfer A. Bagnall A. J. Gray G. A. (2010). Endothelial cell-specific ETB receptor knockout: autoradiographic and histological characterisation and crucial role in the clearance of endothelin-1. Can. J. physiology Pharmacol. 88 (6), 644–651. 10.1139/Y10-041 20628430

[B16] Kuc R. E. Karet F. E. Davenport A. P. (1995). Characterization of peptide and nonpeptide antagonists in human kidney. J. Cardiovasc Pharmacol. 26 (Suppl. 3), S373–S375. 10.1097/00005344-199506263-00111 8587419

[B17] Mager D. E. Krzyzanski W. (2005). Quasi-equilibrium pharmacokinetic model for drugs exhibiting target-mediated drug disposition. Pharm. Res. 22 (10), 1589–1596. 10.1007/s11095-005-6650-0 16180117

[B18] Miyauchi Y. Sakai S. Maeda S. Shimojo N. Watanabe S. Honma S. (2012). Increased plasma levels of big-endothelin-2 and big-endothelin-3 in patients with end-stage renal disease. Life Sci. 91 (13), 729–732. 10.1016/j.lfs.2012.08.008 22921304

[B19] Okada M. Nishikibe M. (2002). BQ-788, A selective endothelin ETB receptor antagonist. Cardiovasc. Drug Rev. 20 (1), 53–66. 10.1111/j.1527-3466.2002.tb00082.x 12070534

[B20] Packer M. McMurray J. J. V. Krum H. Kiowski W. Massie B. M. Caspi A. (2017). Long-term effect of endothelin receptor antagonism with bosentan on the morbidity and mortality of patients with severe chronic heart failure: primary results of the ENABLE trials. JACC Heart Fail. 5 (5), 317–326. 10.1016/j.jchf.2017.02.021 28449795

[B21] Parker J. D. Thiessen J. J. Reilly R. Tong J. H. Stewart D. J. Pandey A. S. (1999). Human endothelin-1 clearance kinetics revealed by a radiotracer technique. J. Pharmacol. Exp. Ther. 289 (1), 261–265. 10087013

[B22] Peter M. G. Davenport A. P. (1996). Characterization of the endothelin receptor selective agonist, BQ3020 and antagonists BQ123, FR139317, BQ788, 50235, Ro462005 and bosentan in the heart. Br. J. Pharmacol. 117 (3), 455–462. 10.1111/j.1476-5381.1996.tb15212.x 8821534 PMC1909322

[B23] Puy A. Lo P. S. Saltelli A. Levin S. A. (2022). Sensobol: an R package to compute variance-based sensitivity indices. J. Stat. Softw. 102 (5), 1–37. 10.18637/jss.v102.i05

[B24] Russell F. D. Davenport A. P. (1996). Characterization of the binding of endothelin ETB selective ligands in human and rat heart. Br. J. Pharmacol. 119 (4), 631–636. 10.1111/j.1476-5381.1996.tb15720.x 8904635 PMC1915765

[B25] Schweizer A. Valdenaire O. Nelböck P. Deuschle U. Dumas Milne Edwards J. B. Stumpf J. G. (1997). Human endothelin-converting enzyme (ECE-1): three isoforms with distinct subcellular localizations. Biochem. J. 328 (Pt 3), 871–877. 10.1042/bj3280871 9396733 PMC1218999

[B26] Strachan F. E. Spratt J. C. Wilkinson I. B. Johnston N. R. Gray G. A. Webb D. J. (1999). Systemic blockade of the endothelin-B receptor increases peripheral vascular resistance in healthy men. 33(1):581–585. 10.1161/01.hyp.33.1.581 9931169

[B27] Vercauteren M. Trensz F. Pasquali A. Cattaneo C. Strasser D. S. Hess P. (2017). Endothelin ET_A_ receptor blockade, by activating ET_B_ receptors. Increases Vasc. Permeability Induces Exaggerated Fluid Retent. 361 (2), 322–333. 10.1124/jpet.116.234930 28223322

[B28] Waijer S. W. Gansevoort R. T. Bakris G. L. Correa-Rotter R. Hou F.-F. Kohan D. E. (2021). The effect of atrasentan on kidney and heart failure outcomes by baseline albuminuria and kidney function: A: *post hoc*: analysis of the SONAR randomized trial. Clin. J. Am. Soc. Nephrol. 16 (12), 1824–1832. 10.2215/CJN.07340521 34853062 PMC8729501

[B29] Wang W. Hallow K. James D. (2016). “A tutorial on RxODE: simulating differential equation pharmacometric models in R,”. Editor CPT R., 5, 3–10. 10.1002/psp4.12052 pharmacometrics and Syst. pharmacology1 PMC4728294 26844010

