# Supplementary material for: Kinetics of endothelin-1 and effect selective ETA antagonism on ETB activation: a mathematical modeling analysis
Source: Front Pharmacol. 2024 Nov 26;15:1332388. doi: 10.3389/fphar.2024.1332388 (PMC11632605; doi:10.3389/fphar.2024.1332388)
Supplement: Supplementary file 1 [file DataSheet1.docx]

Supplementary Material

# Derivation of ET-1 kinetic equations in the presence of a competitive selective endothelin antagonist.

By definition, the dissociation constant K_d_ of ET-1 for its receptors is:

$K_{d}=\frac{[R_{A}]*[ET1]}{[ET1R_{A}]}; K_{d}=\frac{[R_{B}]*[ET1]}{[E{T1R}_{B}]}$ *(A1)*

where [ET] is free ET-1 concentration, [R_A_] and [R_B_] are free receptor concentrations, and [ER_A_] and [ER_B_] are the concentrations of the ligand-receptor complexes.

The binding affinities K_i_ of an endothelin receptor antagonist for its receptors are:

$K_{ia}=\frac{[R_{A}]*[I]}{[IR_{a}]}; K_{ib}=\frac{[R_{B}]*[I]}{[IR_{B}]}$ *(A2)*

Where [I] is the concentration of the antagonist, and [IR_A_] and [IR_B_] are the concentrations of the antagonist-receptor complexes.

Total ET-1 concentration is the sum of free ET-1 and ET-1 bound to each receptor type:

$\left[ R_{A} \right]_{tot}=\left[ R_{A} \right]+\left[ E{T1R}_{A} \right]+[IR_{A}]$ *(A3)*

$\left[ R_{B} \right]_{tot}=\left[ R_{B} \right]+\left[ ET1R_{B} \right]+[IR_{B}]$ *(A4)*

Solving A1 for [R_A_] gives:

$\left[ R_{A} \right]=\frac{K_{d}\left[ E{T1R}_{A} \right]}{\left[ ET1 \right]}$ *(A5)*

Solving A2 for [IR_A_] and plugging in A5 gives:

$\left[ IR_{A} \right]=\frac{K_{d}\left[ ET1R_{A} \right]\left[ I \right]}{\left[ ET1 \right]K_{ia}}$ (A6)

Substituting A5 and A6 into A3 gives:

$\left[ R_{A} \right]_{tot}=\frac{K_{d}\left[ E{T1R}_{A} \right]}{\left[ ET1 \right]}+\left[ ET1R_{A} \right]+\frac{K_{d}\left[ ET1R_{A} \right]\left[ I \right]}{\left[ ET1 \right]K_{ia}}$ *(A7)*

Factoring gives:

$\left[ R_{A} \right]_{tot}=\left[ E{T1R}_{A} \right]\left( \frac{\left[ ET1 \right]+K_{d}+\frac{K_{d}\left[ I \right]}{K_{ia}}}{\left[ ET1 \right]} \right)$ *(A8)*

Solving for [ET1R_A_], the concentration of ET1- bound to the ET_A_ receptor can be determined as a function of receptor concentration, ET-1 concentration, antagonist concentration ([I]) the binding affinities K_d_ and K_ia_.

$\left[ E{T1R}_{A} \right]=\frac{\left[ R_{A} \right]_{tot}\left[ ET1 \right]}{K_{d}\left( 1+\frac{\left[ I \right]}{K_{ia}} \right)+[ET1]}$ (A9)

Analogously, [ER_B_] is given by:

$\left[ E{T1R}_{B} \right]=\frac{\left[ R_{B} \right]_{tot}\left[ ET1 \right]}{K_{d}\left( 1+\frac{\left[ I \right]}{K_{ib}} \right)+[ET1]}$ (A10)

To determine the concentration of unbound ET-1 in the presence of endothelin antagonists, first, again rearrange A1 and substitute into A3:

$\left[ R_{A} \right]_{tot}=\left[ R_{A} \right]+\frac{\left[ R_{A} \right][ET1]}{K_{d}}+\frac{\left[ R_{A} \right][I]}{K_{ia}}$ *(A11)*

Then, the free receptor concentration [R_A_] is:

$\left[ R_{A} \right]=\frac{\left[ R_{A} \right]_{tot}}{1+\frac{\left[ ET1 \right]}{K_{d}}+\frac{\left[ I \right]}{K_{ia}}}$ *(A12)*

And analogously:

$\left[ R_{B} \right]=\frac{\left[ R_{B} \right]_{tot}}{1+\frac{\left[ ET1 \right]}{K_{d}}+\frac{\left[ I \right]}{K_{ib}}}$ *(A13)*

Total ET-1 is the sum of free ET-1 and ET-1 bound to the ET_A_ and ET_B_ receptors:

$[E{T1]}_{tot}=\left[ ET1 \right]+\left[ E{T1R}_{A} \right]+\left[ ET1R_{B} \right]$ *(A14)*

Substituting A12 and A13 into A 14 gives:

$[E{T1]}_{tot}=\left[ ET1 \right]+\frac{\left[ ET1 \right]}{K_{d}}\left( \frac{\left[ R_{A} \right]_{tot}}{1+\frac{\left[ ET1 \right]}{K_{d}}+\frac{\left[ I \right]}{K_{ia}}}+\frac{\left[ R_{B} \right]_{tot}}{1+\frac{\left[ ET1 \right]}{K_{d}}+\frac{\left[ I \right]}{K_{ib}}} \right)$ *(A15)*

Multiplying this out and rearranging gives a 3^rd^ order polynomial in terms of free ET-1:

$-\frac{\left[ ET1 \right]^{3}}{K_{d}^{2}}+\left[ ET1 \right]^{2}\left( \frac{[E{T1]}_{tot}}{K_{d}^{2}}-\frac{2}{K_{d}}- \frac{\left[ I \right]}{K_{d}K_{ib}}- \frac{\left[ I \right]}{K_{d}K_{ia}}- \frac{1}{K_{d}^{2}}\left( \left[ R_{A} \right]_{tot}+\left[ R_{B} \right]_{tot} \right) \right)+\left[ ET1 \right]\left( \frac{2[E{T1]}_{tot}}{K_{d}}+[E{T1]}_{tot}\frac{\left[ I \right]}{K_{d}K_{ia}}+[E{T1]}_{tot}\frac{\left[ I \right]}{K_{d}K_{ib}} -\left( 1+ \frac{\left[ I \right]}{K_{ia}}+\frac{\left[ I \right]}{K_{ib}}+ \frac{\left[ I \right]^{2}}{K_{ib}K_{ia}} \right)- \frac{1}{K_{d}}\left( \left[ R_{A} \right]_{tot}\left( 1+\frac{[I]}{K_{ib}} \right)+\left[ R_{B} \right]_{tot}\left( 1+\frac{[I]}{K_{ia}} \right) \right) \right)+ [E{T1]}_{tot}\left( 1+\frac{\left[ I \right]}{K_{ia}}+ \frac{\left[ I \right]}{K_{ib}}+ \frac{\left[ I \right]^{2}}{K_{ib}K_{ia}} \right)=0$ *(A16)*

This cubic equation can then be solved to determine free ET-1.


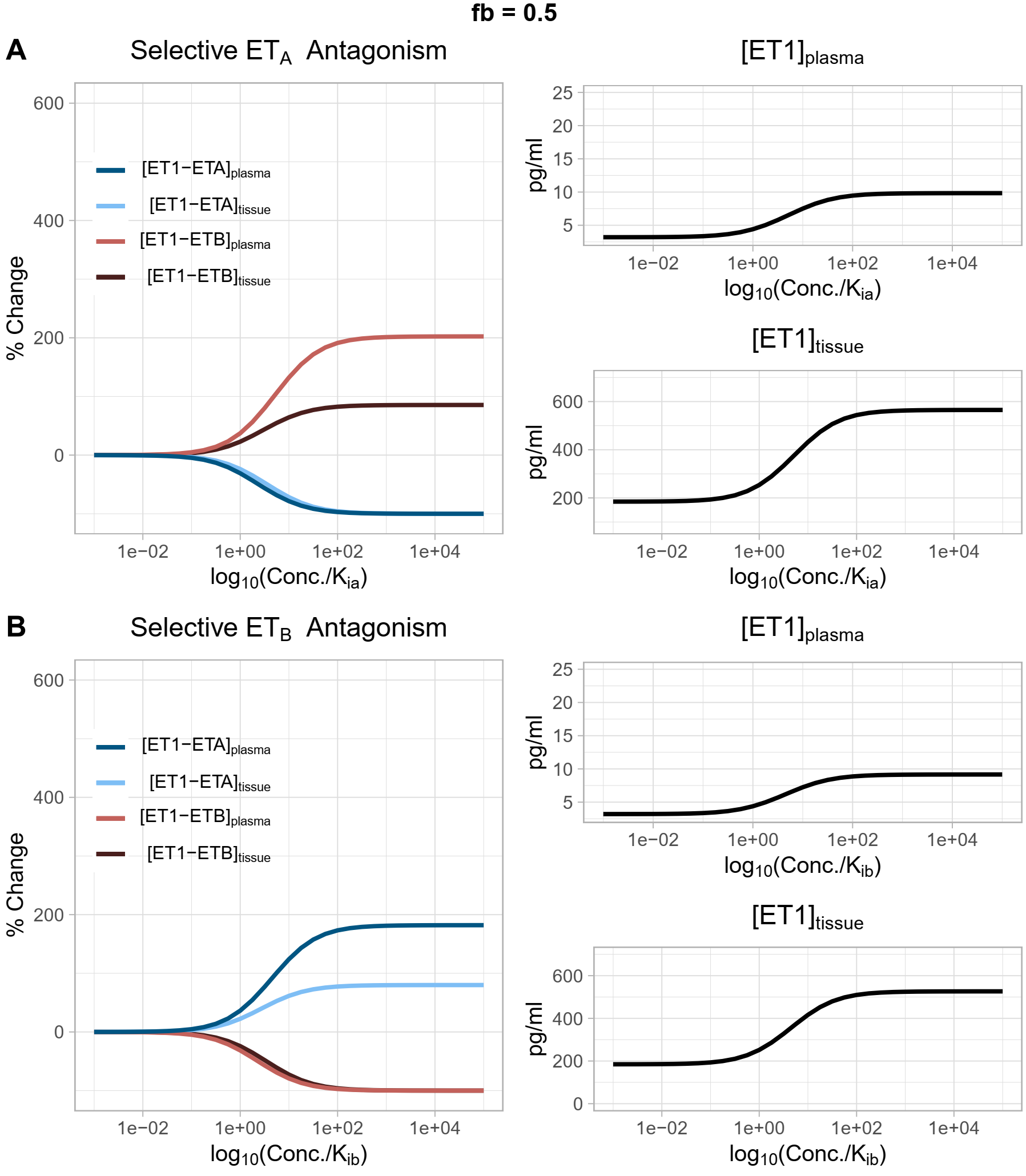


**Supplementary Figure 1.** Simulation presented in Figure 4 repeated but with f_b_ (fraction of total receptors that are ET_B_ receptors) set to 0.5 instead of estimated value. The rise in the non-antagonized receptor complex was equal for selective ET_A_ and ET_B_ antagonists (i.e. ET1-ET_B_ rise with ET_A_ antagonism was the same as ET1-ET_A_ rise with ET_B_ antagonism). The ET-1 concentration also rose equally. The shape of the curves, and thus the dependency on K_i_ and concentration, remained the same as in Figure 4. Only the magnitudes changed.


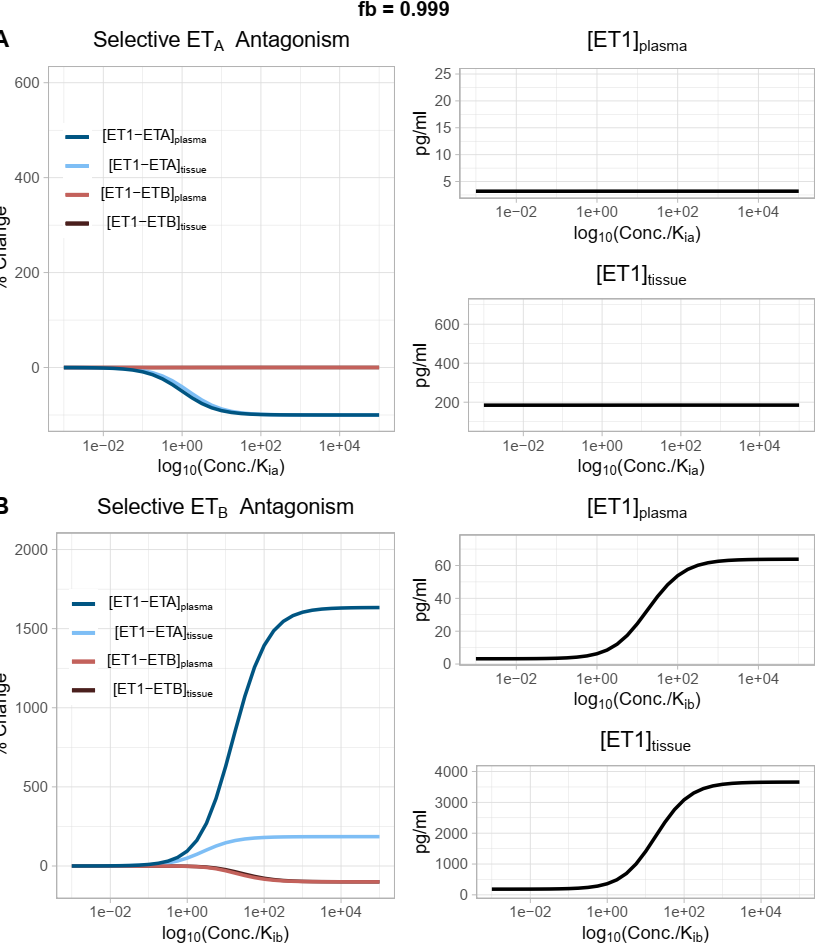


**Supplementary Figure 2. Simulation presented in Figure 4 repeated but with f_b_ (fraction of total receptors that are ET_B_ receptors) set to 0.999 instead of estimated value.** There was no change in ET1-ET_B_ with ET_A_ antagonism, but ET1-ET_A_ increased more than 2000-fold with ET_B_ antagonism. The shape of the curves, and thus the dependency on K_i_ and concentration, remained the same as in Figure 4. Only the magnitudes changed.
